# Central serous chorioretinopathy following oral quetiapine

**DOI:** 10.3205/oc000221

**Published:** 2023-07-12

**Authors:** Ceren Durmaz Engin, Mehmet Orcun Akdemir

**Affiliations:** 1 Department of Ophthalmology, Karadeniz Eregli State Hospital, Zonguldak, Turkey; 2 Department of Ophthalmology, Bulent Ecevit University School of Medicine, Zonguldak, Turkey

**Keywords:** atypical antipsychotic, central serous chorioretinopathy, quetiapine, retina

## Abstract

Central serous chorioretinopathy (CSCR) is a chorioretinal disease that is characterized by central vision loss and is usually seen in middle-aged males. It has been associated with the use of various drugs, including corticosteroids and phosphodiesterase inhibitors. We present the case of a 36-year-old male who developed CSCR after a few weeks of irregular use of quetiapine for his sleep problems. The clinical findings of the patient improved shortly after he stopped using the drug, and at the end of the two-month period complete recovery was observed. Quetiapine is an atypical antipsychotic that exerts its effects on dopamine and serotonin receptors. There are studies showing that these neurotransmitters may play a role in the control of choroidal vascular permeability, which is the underlying cause of CSCR. Therefore, we believe that quetiapine may have a causal relationship with CSCR. To our knowledge, this is the first case report documenting full recovery from quetiapine associated CSCR.

## Introduction

Central serous chorioretinopathy (CSCR) is a chorioretinal disease which usually affects the central macula and is characterized by subretinal fluid accumulation leading to acute or sub-acute vision loss, metamorphopsia, and a hyperopic shift. It is most prevalent in males between the ages of 20 and 50. Although the pathophysiological mechanism of the disease has not yet been fully elucidated, CSCR is thought to arise from hyperpermeable choroidal capillaries that cause a serous detachment of the neurosensory retina [1]. Corticosteroids, phosphodiesterase inhibitors, pseudoephedrine, mefloquine, and pregabalin have all been linked to CSCR in previous studies [2], [3].

Quetiapine is an atypical antipsychotic drug that works by acting on serotonin and dopamine receptors to alleviate the positive and negative symptoms of schizophrenia and severe bipolar disease. In recent years, it is increasingly used off-label for the treatment of sleep disturbances or insomnia [4]. Although ocular side effects are rare, atypical antipsychotic drugs may cause blurred vision, cataract, oculogyric crises, seeing halos around lights, and temporary myopia [5], [6]. In this case report, we present a male patient who developed CSCR after off-label use of quetiapine for his sleep problems.

## Case description

**Figure 1:**
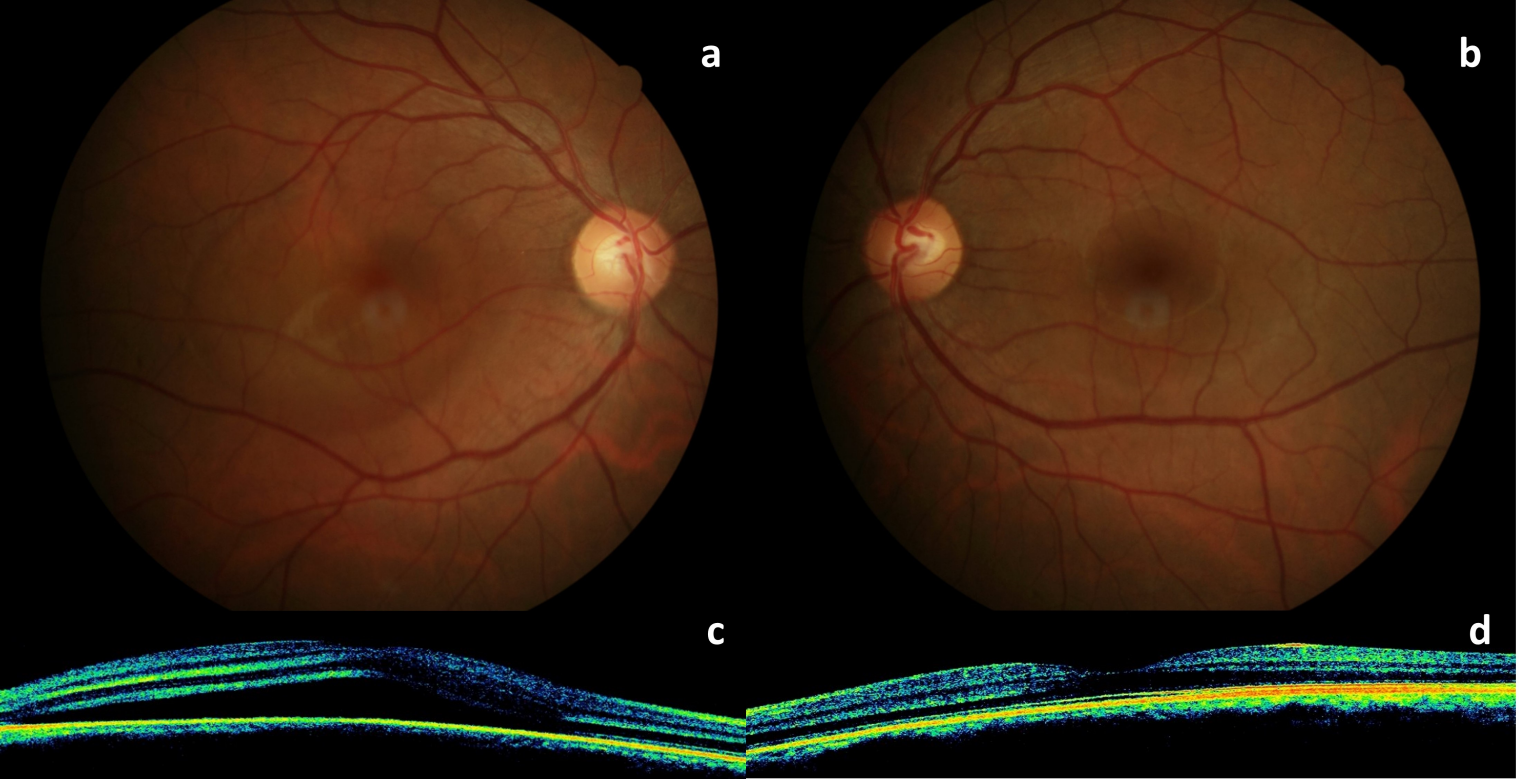
a: color fundus photo of the right eye showing serous retinal detachment approximately four disc diameter in size in the macula; b: color fundus photo of a normal looking left eye; c: SD-OCT of the right eye shows a large area of subretinal fluid; d: SD-OCT of the left eye is normal

**Figure 2:**
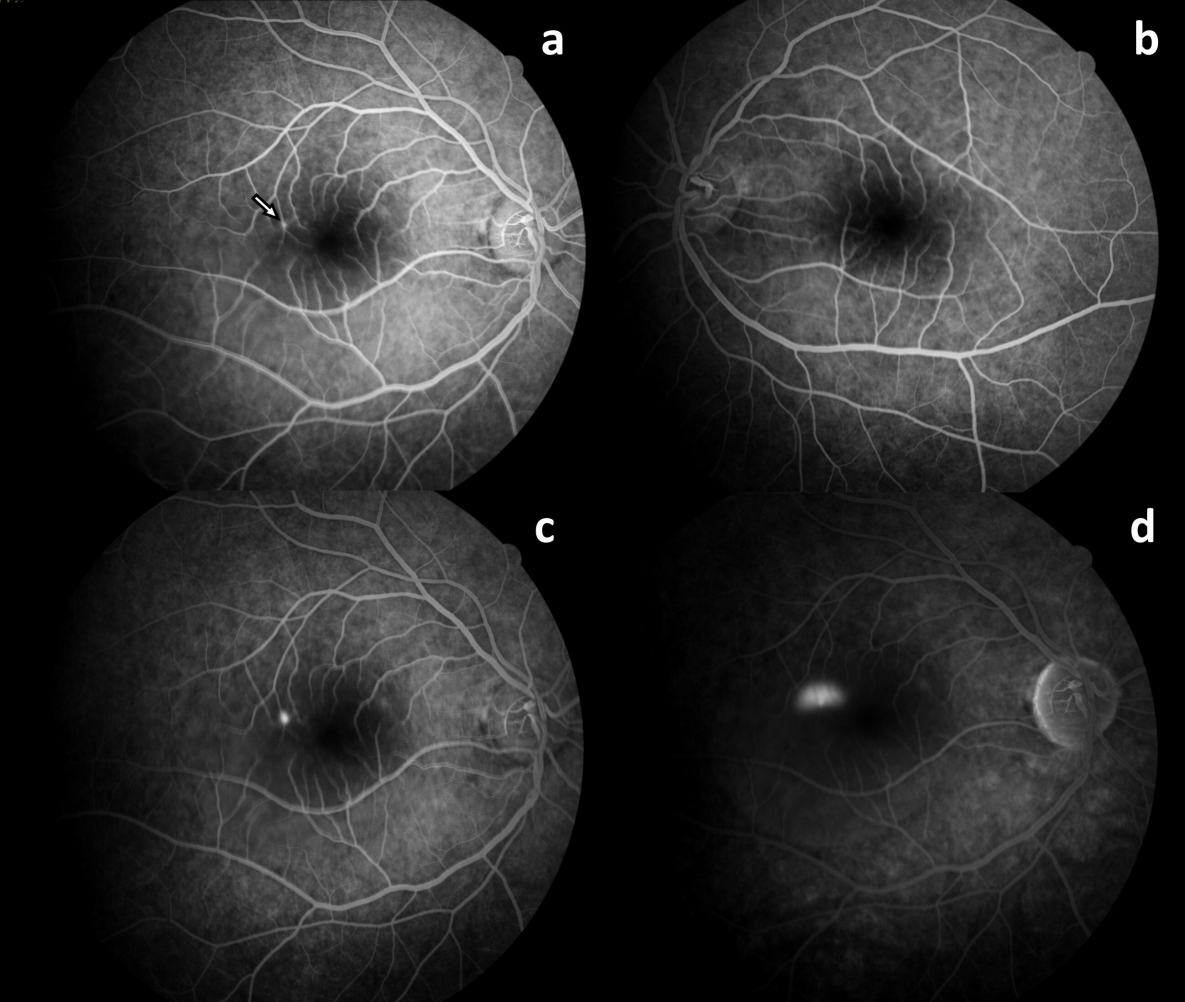
a: FA of the right eye in the early phase shows a hyperfluorescent spot (arrow); b: FA of the left eye is normal; c and d: mid-to-late phases of FA showing expansion of hyperfluorescence in an inkblot pattern in the right eye

**Figure 3:**
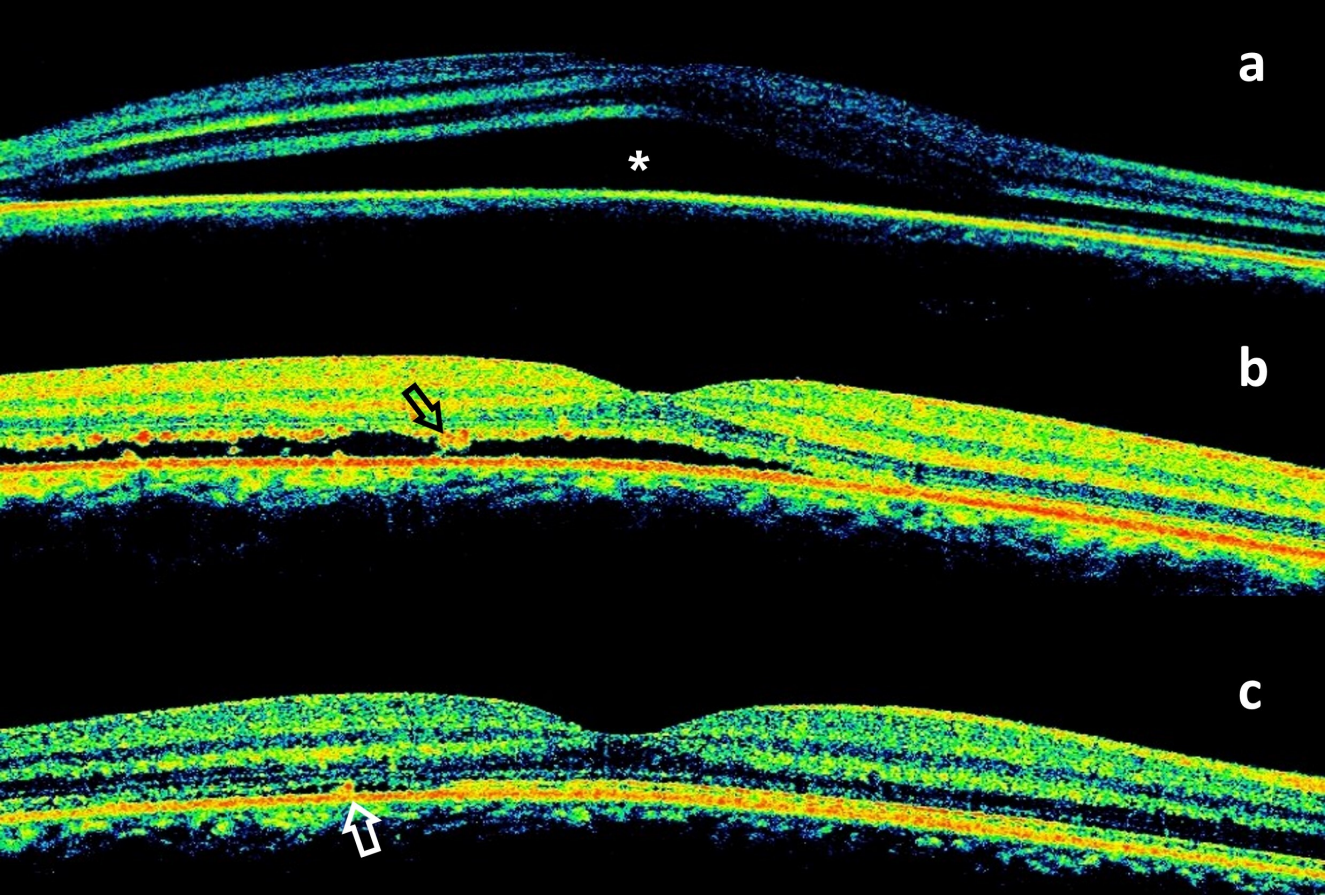
a: SD-OCT of the right eye shows a large area of subretinal fluid (*); b: resolution of subretinal fluid and brush-border-like appearance (black arrow) in follow-up OCT; c: a flat-appearing macula with a small residual subretinal fluid and minor RPE changes (white arrow)

A 36-year-old male patient was admitted to our clinic with the complaint of decreased central vision in his right eye for two weeks. He had no known history of systemic or ocular diseases. He had been taking quetiapine (Gyrex 100 mg, Santa Farma, Turkey) off-label for the past few weeks for his sleep disturbance. He stated that he took one or two pills occasionally before going to sleep and that his vision problems began after taking this medication. He had no other addictions. Best corrected visual acuity (BCVA) on eye examination was 20/30 and 20/20 in the right (OD) and left eye (OS), respectively. Slit lamp examination findings and intraocular pressure were normal in both eyes. Fundus examination revealed a large serous retinal detachment approximately four disc diameter in size in OD (Figure 1a (Fig. 1)). Spectral domain optical coherence tomography (SD-OCT) showed subretinal fluid in the macula (Figure 1c (Fig. 1)). Central foveal thickness (CFT) was 513 µm in OD and 261 µm in OS. Fluorescein angiography (FA) showed the classical inkblot pattern with expanding hyperfluorescence in OD (Figure 2a, c, d (Fig. 2)). Fundoscopy, SD-OCT, and FA findings in OS were normal (Figure 1b, d (Fig. 1) and Figure 2b (Fig. 2)). The patient was asked to discontinue quetiapine. Two weeks later, his BCVA was 20/20 in OD and there was a marked reduction in the extent of the lesion in OCT. CFT was measured as 309 µm, and there was brush-border-like appearance in OD (Figure 3b (Fig. 3)). At the follow-up examination 2 months later, the serous elevation was almost completely resolved, and OCT revealed a flat-appearing macula with minor retina pigment epithelium (RPE) changes with a CFT of 247 µm (Figure 3c (Fig. 3)).

## Discussion

It was previously believed that RPE dysfunction was the major cause of CSCR, but new imaging methods reveal that RPE dysfunction may actually be a consequence of CSCR rather than its cause [7], [8]. Choroidal vascular hyperpermeability, delay in vascular filling, and venous congestion in choroidal vessels revealed by indocyanine green angiography (ICGA) studies suggest an abnormal choroidal blood flow in patients affected by CSCR [9]. Neuromodulation of choroidal vessels by corticosteroids may explain the well-known relationship between hypercortisolism and CSCR. Increased catecholamine release and potentiation of their effects in the choroidal vasculature by corticosteroids could also contribute to CSCR [10]. Dopamine and serotonin are other neurotransmitters that have a role in ocular circulation [11], [12].

Quetiapine is a second-generation antipsychotic that has an affinity for D2, 5-HT2A, H1, alpha 1, and 5-HT1A receptors. Although low, quetiapine has been shown to have affinity for excitatory D1 receptors which may modulate dopaminergic vasodilation in the choroidal circulation [11]. Animal studies about the role of dopamine in the control of ocular blood flow are controversial. Dopamine antagonists have been shown to increase ocular blood flow in rabbits [13]. Another study showed dopamine causes a dose-dependent effect on ciliary blood flow, with ciliary vasodilation at the lowest infusion rate and vasoconstriction at higher infusion rates [14]. In a study investigating the effect of dopamine on choroidal blood flow in human subjects, fundus pulsation amplitude was found to be regulated by dopamine, but its effect on choroidal blood flow has not been fully explained [15].

Quetiapine exerts its serotonergic activity by 5-HT2A receptor antagonism and partial agonism of 5-HT1A receptors. Hayreh et al. [12] found that in atherosclerotic monkey eyes serotonin can cause temporary occlusion of the posterior ciliary artery (PCA) supplying the choroid, but this effect is not seen in non-atherosclerotic monkey eyes. On the other hand, serotonin-mediated ciliochoroidal effusion has been proposed as a mechanism for transient myopia associated with atypical antipsychotics, including quetiapine and aripiprazole [6]. These different effects may be explained by the findings of another study that, while serotonin induces vasoconstriction by 5-HT2C and 5-HT2B receptors in PCA, in bovine eyes it has been observed that it does not exert a contractile effect by 5-HT1A and 5-HT3 receptors [16].

Despite the lack of consensus on the results, dopamine and serotonin appear to have effects on the regulation of ocular and choroidal blood flow, and quetiapine may modulate choroidal vasculature via these neurotransmitters.

Although ours is the second case in the literature to report the occurence of CSCR after quetiapine use, it is the first case to document complete recovery from the disease. Jain reported a case of recurrent CSCR following quetiapine therapy in a 30-year-old insomniac patient [17]. Although this patient also had a history of steroid use, the author hypothesized a relation between quetiapine and CSCR due to clinical improvement in a short time after discontinuation of quetiapine and recurrence after repeated quetiapine use.

We did not have access to enhanced depth imaging OCT or ICGA in this patient to examine choroidal thickness or vascular alterations in the choroid. However, we think that the traditional OCT and FA findings are adequate to diagnose the patient with CSCR. Furthermore, while the patient might not mention a recent increase in his regular stress level, sleep issues may be a sign of increased stress. However, a temporal relation to quetiapine intake, as well as improvement in serous elevation after two weeks following withdrawal, suggests a possible causative relation between quatiapine and CSCR.

## Conclusion

Quetiapine may cause CSCR by affecting choroidal blood flow via dopamineric and serotonergic pathways. In individuals who have reduced visual acuity during quetiapine treatment, a retinal examination is necessary to rule out the possibility of CSCR.

## Notes

### Informed consent 

Informed consent has been obtained from the patient for the publication of this case report. 

### Authors’ ORCIDs

- Ceren Durmaz Engin, M.D.: 0000-0001-5797-6467

### Competing interests

The authors declare that they have no competing interests.
